# Engineering transcription factor-based biosensors for repressive regulation through transcriptional deactivation design in *Saccharomyces cerevisiae*

**DOI:** 10.1186/s12934-020-01405-1

**Published:** 2020-07-20

**Authors:** Chenxi Qiu, Xiaoxu Chen, Reheman Rexida, Yu Shen, Qingsheng Qi, Xiaoming Bao, Jin Hou

**Affiliations:** 1 State Key Laboratory of Microbial Technology, Shandong University, Binhai Road 72, Qingdao, 266237 People’s Republic of China; 2 State Key Laboratory of Biobased Material and Green Papermaking, School of Bioengineering, Qi Lu University of Technology, Jinan, 250353 People’s Republic of China; 3 Present Address: State Key Laboratory of Natural and Biomimetic Drugs, School of Pharmaceutical Sciences, Peking University, Beijing, 100191 People’s Republic of China

**Keywords:** Biosensors, *Saccharomyces cerevisiae*, Repressive regulation, Med2

## Abstract

**Background:**

With the development of engineering the microbial cell factories, biosensors have been used widely for regulation of cellular metabolism and high-throughput screening. However, most of the biosensors constructed in *Saccharomyces cerevisiae* are designed for transcriptional activation. Very few studies have dedicated to the development of genetic circuit for repressive regulation, which is also indispensable for the dynamic control of metabolism.

**Results:**

In this study, through transcriptional deactivation design, we developed transcription-factor-based biosensors to allow repressive regulation in response to ligand. Using a malonyl-CoA sensing system as an example, the biosensor was constructed and systematically engineered to optimize the dynamic range by comparing transcriptional activity of the activators, evaluating the positions and numbers of the operators in the promoter and comparing the effects of different promoters. A biosensor with 82% repression ratio was obtained. Based on this design principle, another two biosensors, which sense acyl-CoA or xylose and downregulate gene expression, were also successfully constructed.

**Conclusions:**

Our work systematically optimized the biosensors for repressive regulation in yeast for the first time. It provided useful framework to construct similar biosensors. Combining the widely reported biosensors for transcriptional activation with the biosensors developed here, it is now possible to construct biosensors with opposing transcriptional activities in yeast. 
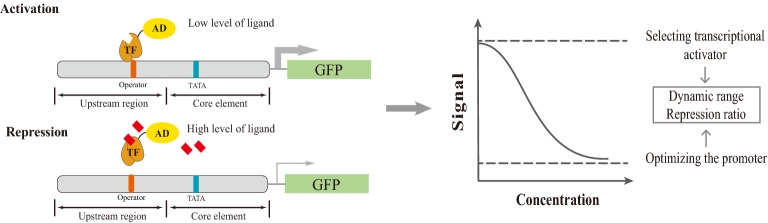

## Background

Metabolic engineering has been applied to produce a wide variety of valuable products, including biofuels, bulk chemicals, fine chemicals, nutraceuticals, and pharmaceuticals. So far, it is remaining a challenge to achieve precise regulation of metabolic network. Genetically-encoded biosensors provide useful tools for dynamic control of biosynthesis pathway [1, 2]. Biosensor is a molecular device that can sense molecules of interest and output a detectable signal in response. It can either derive from transcription factors (TFs) or from RNA devices. TFs that respond to changes in metabolites and alter the transcription of genes have been widely used as metabolite biosensors. Increasing numbers of prokaryotic TFs have recently been successfully transferred into yeast to develop biosensors [3–10]. However, most of the biosensors constructed in *S. cerevisiae* have been designed to activate gene expression in response to an increase in a metabolite concentration, whereas very few studies have dedicated to developing repressive regulation system in *S. cerevisiae*, perhaps because the dynamic regulation in yeast was not studied as widely as in prokaryotic organisms [11, 12].

Similar to biosensor for transcriptional activation, the genetic circuit for repressive regulation is also indispensable to dynamically control a metabolic pathway or screen for high-producer cells. For instance, it was reported that two malonyl-CoA biosensors that had either activating or repressive transcriptional activities were constructed to dynamically regulate fatty acid biosynthesis synergistically in *Escherichia coli* [13]. In their design, repressive regulation was used to control the transcription of the malonyl-CoA synthesis pathway and activating regulation was used to control the transcription of the malonyl-CoA sink pathway. In prokaryotes, it is also possible to obtain repressive regulation when combining multiple activating TF-promoter systems. For example, P_tet_ and P_lac_ was combined to create an inverting gene circuit, and used to downregulate glucokinase to improve gluconate yields in *E. coli* [14]. To screen for high-producer cells, a biosensor to switch off the expression of toxic protein with the increase of the metabolites level was often required to generate a growth-based selection system [15].

To date, only a few TFs such as tetR [16] and BenM [17] were engineered to construct the regulation system for transcriptional repression in *S. cerevisiae*. Tetracycline-inducible activator and repressor were based on tetR and a mutated tetR moiety to fuse with eukaryotic activator or repressor to create the regulation system. BenM was engineered by directed evolution to reverse the function of a cis,cis-muconic-acid-inducible biosensor from activation to repression. However, engineering such biosensor requires protein engineering, which is a labor-consuming process. A common strategy to construct the TF-based biosensor for transcriptional activation is to express an allosteric TF and inserting its operators into the promoter. When TF binds to the promoter, the steric hindrance inhibits the binding of transcriptional machinery, and thereby blocks transcription. With an increase in the level of ligand, the dissociation of TF from the promoter leads to the transcriptional activation. The biosensors that can sense metabolites such as malonyl-CoA [3–6, 13], xylose [18, 19], acyl-CoA [20, 21], and fructose-1,6-bisphosphate [22] were designed based on this principle.

**Fig. 1:**
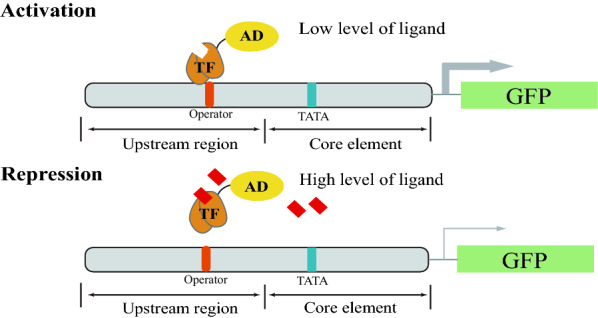
Design of the repressive biosensor through transcriptional deactivation in response to an increase in ligand. The transcription factor (TF) was fused to the activation domain (AD) of the transcriptional activator. Contrary to the biosensor for transcriptional activation which operator was inserted into the core promoter close to the TATA box, the operators of the TF were inserted into the upstream region of the promoter to design the biosensor for transcriptional repression. When the ligand concentration was low, TF-AD binded to the operator and recruited the transcription machinery to activate the expression of an output signal (GFP). When the ligand concentration increased, TF-AD released from the operator and deactivated the expression of GFP

In this study, the transcription deactivation strategy was used to develop the biosensor to allow transcriptional repression. Different from the above design, TF was fused with transcriptional activation domain (AD) of an activator to activate the transcription when it binds to the promoter (Fig. 1). In the presence of ligand, it deactivates the transcription. In eukaryotic organisms, activator can stimulate the transcription by recruiting the components in transcriptional machinery such as the TFIID complex or Mediator. They can also alter the chromatin structure to free up DNA-binding sites to facilitate the transcription [23]. Fusion of AD to DNA binding domain (BD) or a transcription factor from prokaryotic organism can create a synthetic activator. Our design is based on the transcription deactivation design (Fig. 1). Using an extensively studied FapR/*fapO* system as an example, we constructed the biosensor and optimize the dynamic range systematically. The design principle was successfully used to construct another two biosensors, which repressed transcription in response to acyl-CoA or xylose. Our study has provided a framework to rationally engineer biosensor for repressive regulation.

## Results

### Construction of the malonyl-CoA repressive biosensor in *S. cerevisiae* and evaluation of the activation efficiency of different ADs in biosensor

**Fig. 2:**
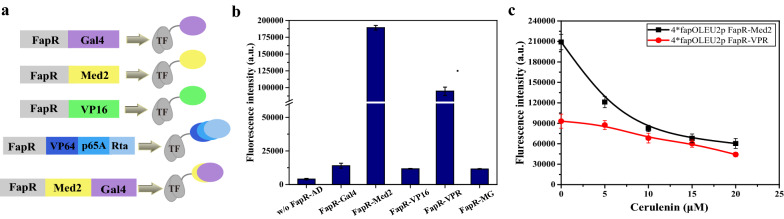
Comparison of different activators in the malonyl-CoA repressive sensor. **a** Schematic design of FapR-AD. **b** Fluorescence intensity of the recombinant strains with different activators. **c** Dose–response curves of FapR-VPR and FapR-Med2 containing strains in the presence of different concentrations of cerulenin. The data are presented as the means ± standard deviations from three independent experiments

In TF-based biosensor for transcriptional activation, TF binds to its operator in the promoter to inhibit transcription. The operator is generally placed in the core promoter close to TATA box or transcriptional start site [24]. In contrast, in order to design TF-based biosensor for transcriptional repression, TF is fused with AD and acts as a transcriptional activator in the absence of ligand, and the binding of TF to promoter activates transcription. In this case, TF is fused with AD to create a synthetic activator (Fig. 1). Its operator is therefore placed in the upstream region of the promoter and acts as an enhancer. With an increase in the level of ligand, TF-AD is dissociated from the promoter to deactivate the transcription, and thereby down-regulate gene expression. We used malonyl-CoA sensing FapR/*fapO* [25] to construct the repressive biosensor in *S. cerevisiae*. In the initially design, FapR from *Bacillus subtilis* was fused to Gal4 AD, a classic AD in yeast, and used as a transcriptional activator. The FapR-binding site *fapO* is a 17-bp DNA sequence and four *fapO* sites were placed 21 bp upstream of the *LEU2* promoter to control the expression of green fluorescent protein (GFP) [26]. As shown in Fig. 2, the expression of FapR-Gal4 increased the transcription by 3.5-fold, demonstrating that FapR-Gal4 can activate the transcription.

Although the activator is an important element affecting the dynamic range of a biosensor, they have not been systematically analyzed in *S. cerevisiae*. Here, we evaluated the activation efficiency of several different activators in the FapR-AD system. Beside yeast Gal4 AD, herpes simplex virus VP16 and the yeast transcriptional mediator Med2 were first evaluated (Fig. 2a). VP16 is a frequently used AD in eukaryotic cells [16, 27]. The subunit of the RNA polymerase II mediator complex is also reported to activate gene expression. Among the different subunits of this mediator, Med2 showed the best activation efficiency [28], so it was also selected as a candidate activator. The fluorescence intensity of the strain containing FapR-VP16 increased 2.9-fold relative to that of the control strain (without FapR-AD) (Fig. 2b). Surprisingly, the strain containing FapR-Med2 showed a much greater increase, and the fluorescence intensity was 46.4-fold higher than the fluorescence intensity of the control strain (without FapR-AD) (Fig. 2b). We also fused FapR with two hybrid activators Med2-Gal4 (MG) and tripartite activator VP64-p65-Rta (VPR) (Fig. 2a). The fluorescence intensity of the strain containing FapR-VPR was 23.2-fold higher than that of the control strain (without FapR-AD), whereas the fluorescence intensity of the strain containing FapR-MG was only 2.9-fold higher than that of the control strain. VPR is reported to be a highly efficient tripartite activator [29], and interestingly, we found that the activation efficiency of FapR-Med2 was even better than that of FapR-VPR in *S. cerevisiae*.

In our design, when the level of ligand increases, TF-AD is dissociated from the promoter to deactivate the transcription, and thereby down-regulate gene expression. Because the biosensors containing FapR-Med2 and FapR-VPR showed the best activation efficiency, the repression ratios of these two biosensors for malonyl-CoA were then evaluated. Cerulenin inhibits fatty acid synthesis by the specific inhibition of β-ketoacyl-acyl carrier protein synthetase [30], and inhibits malonyl-CoA consumption for fatty acid synthesis. Therefore, adding different concentrations of cerulenin leads to the accumulation of malonyl-CoA to different levels. As shown in Fig. 2c, with the graduated addition of cerulenin, the fluorescence intensity decreased gradually, and the saturation concentration was about 20 µM. Relative to the situation without cerulenin, the fluorescence intensity of the strain expressing FapR-Med2 decreased by 72% and the fluorescence intensity of the strain expressing FapR-VPR decreased by 52% with the addition of 20 µM cerulenin. These results clearly show that compared with other frequently used activators, Med2 has much better activation efficiency and allows the FapR-AD repressive biosensor system to sense malonyl-CoA within a broader dynamic output range (the ratio of GFP expression in the absence of cerulenin and in the presence of saturated concentration of cerulenin).

### Optimizing the promoter to expand the dynamic output range of biosensor

**Fig. 3:**
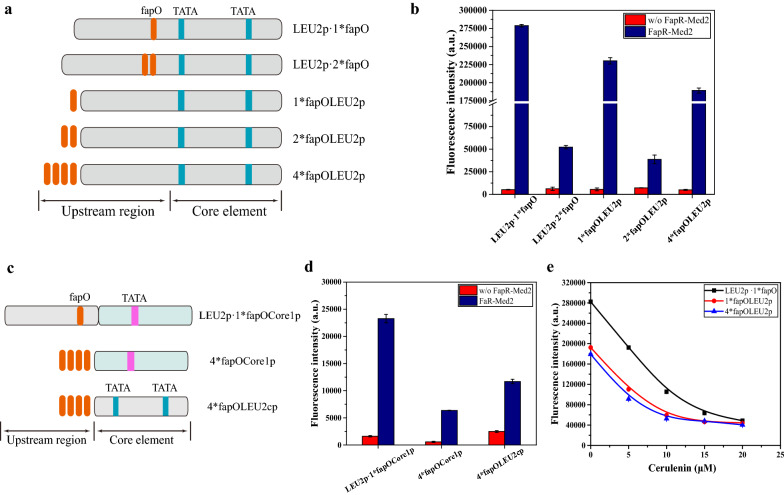
Effects of the position and number of operators in different promoters. **a** Schematic design showing the positions and numbers of *fapO* site in the *LEU2* promoter. **b** Different numbers and locations of the *fapO* site in the *LEU2* promoter were evaluated. **c** Schematic design showing the positions and numbers of *fapO* site in the *LEU2* core promoter and the synthetic minimal core promoter *Core1*. **d** Full-length *LEU2* promoter was replaced with the *LEU2* core promoter (*LEU2cp*) and the synthetic minimal core promoter (*Core1p*) and the effects were compared. **e** Dose–response curves of LEU2p·1*fapO, 1*fapOLEU2p, and 4*fapOLEU2p containing strains in the presence of different concentrations of cerulenin. The data are presented as the means ± standard deviations from three independent experiments

To achieve efficient gene activation, aside from activator, the promoter characteristics also needs to be optimized. The promoter strength and the number and positions of the operators in the promoter were optimized in this study. Here, the weak *LEU2* promoter was used as the basic promoter because we wanted to achieve low expression when it was completely deactivated. For transcription activation, *fapO* sites were placed in the upstream region of the promoter to allow FapR-AD to recruit the transcriptional machinery. We systematically analyzed the activation effect of the numbers and positions of the operators in the promoter. The design was as follows (Fig. 3a): one, two, or four *fapO* sites were inserted 21 bp upstream of the *LEU2* promoter, and the distance to the upstream TATA box was 131 bp; or one or two *fapO* sites were inserted inside the upstream region of the promoter, located 51 bp upstream of core element of promoter and the distance to the upstream TATA box was 57 bp. Interestingly, we found that the number of *fapO* sites was not a key factor affecting transcriptional activation. As the activation efficiency did not increase with increased *fapO* sites. The strains containing either one or four *fapO* sites upstream of the *LEU2* promoter or one site inside the promoter showed high activation efficiencies, whereas the strains containing two *fapO* sites upstream of the *LEU2* promoter or inside the promoter showed relatively low activation efficiencies (Fig. 3b). We speculated that because Med2 is a very strong activator, one FapR-Med2 moiety binding to the promoter may be efficient enough to recruit the transcriptional machinery and activate transcription. However, when FapR-Med2 binds to two close operators, the activation efficiency may be weakened by steric hindrance. In contrast, the position of the binding site seems to be important for gene activation. As we can see that the activation efficiency of the strain with one *fapO* site inside the promoter was 22% higher than that of the strain with one site upstream from the *LEU2* promoter (Fig. 3b). We found that among these designs, the strains with one *fapO* site inside the promoter or one or four *fapO* sites inserted 21 bp upstream of *LEU2* promoter had relatively better activation efficiencies, and their fluorescence intensities were 53.5-fold, 41.9-fold, and 38.1-fold higher than that of the control strain (without FapR-AD), respectively.

We also attempted to reduce the basic expression level of the biosensor (the expression level without FapR-AD). Although the *LEU2* promoter is a weak promoter, to further reduce the expression from it, two core promoters (the *LEU2* core promoter and a synthetic minimal core promoter *Core1p*) [31], were used as the basic promoters. Generally, the core promoter only contains the necessary elements for basic transcription, and has a relative low expression than full length of the promoter [32]. A series of synthetic minimal core promoter were created and they showed lower expression than endogenous core promoter [31], thereby synthetic minimal core promoter was also chosen here. Four *fapO* sites were inserted upstream of the core promoters to construct 4*fapOCore1p and 4*fapOLEU2cp. Because one *fapO* site inserted into the upstream region of the *LEU2* promoter showed the best activation efficiency, this upstream region (LEU2p·1*fapO) was also fused with *Core1p* to construct LEU2p·1*fapOCore1p (Fig. 3c). As shown in Fig. 3d, the basic expression from these three designs was always lower than the basic expression from the full-length *LEU2* promoter. Among these constructs, 4*fapOCore1p showed the lowest basic expression level, which was only 14% of that of the full-length *LEU2* promoter. However, its activation efficiency was also lower than that of the full-length *LEU2* promoter. The fluorescence intensity of the strains containing LEU2p·1*fapOCore1p, 4*fapOCore1p, or 4*fapOLEU2cp increased 14.5-fold, 11.1-fold, or 4.7-fold, respectively, when expressing FapR-Med2. We speculated that the activation efficiency may be related to the characteristics of the promoter and to the spatial location of the operator.

We then analyzed the repression ratio in response to the malonyl-CoA level in the strains with best activation efficiency. As shown in Fig. 3e, the fluorescence intensity decreased by 82% when 20 µM cerulenin was added to the strain containing one *fapO* site inside the promoter, and the fluorescence decreased by 79% or 74% in the strain containing either one or four *fapO* sites upstream of the *LEU2* promoter, respectively. In these designs, one *fapO* site inserted into the upstream region of the *LEU2* promoter showed better transcriptional activation and a better dose-dependent repressive response to malonyl-CoA.

From these results, we inferred that the activation efficiency of the activator and the position of the operator in the promoter are the key factors affecting the dynamic range of the response of a repressive biosensor.

### Construction of the fatty acyl-CoA and xylose repressive biosensor

**Fig. 4:**
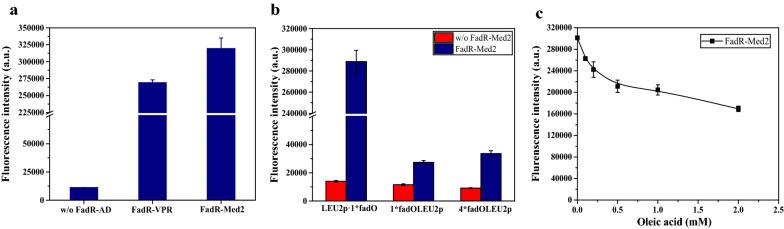
Testing selected designs to construct a fatty acyl-CoA repressive biosensor. **a** Characterization of the transcriptional activity of FadR-VPR and FadR-Med2 in the fatty acyl-CoA biosensor. **b** Characterization of the transcriptional activity of strains containing LEU2p·1*fadO, 1*fadOLEU2p, or 4*fadOLEU2p with FadR-Med2. **c** Dose–response curve of the strain containing LEU2p·1*fadO and FadR-Med2 in the presence of oleic acid (C18:1). The data are presented as the means ± standard deviations from three independent experiments

To test the generality of the biosensor design in engineering other TF-based metabolite-repressive biosensors, we selected two other allosteric TFs, FadR from *E. coli* [33] and XylR from *Staphylococcus xylosus* [19], which respond to fatty acyl-CoA and xylose, respectively, to construct repressive biosensors. In the design of the acyl-CoA-repressing biosensor, VPR and Med2 were fused with FadR and the activation effect was tested. Consistent with the results reported above, the transcriptional activation efficiency of FadR-Med2 (28.4-fold higher than the control) was higher than that of FadR-VPR (23.9-fold higher than the control) (Fig. 4a). To construct the promoters, three designs that have relatively good transcription activity were chosen: one binding site inside the upstream region, and one or four *fapO* inserted 21 bp upstream of the *LEU2* core promoter. The *fapO* sequence was directly replaced with the *fadO* sequence. Consistent with the FapR/*fapO* design, the strain with one *fadO* site inside the upstream region of the *LEU2* promoter also showed the highest transcriptional activation (Fig. 4b). We next evaluated whether the biosensor could downregulate gene expression in response to the addition of oleic acid (C18:1). As expected, the fluorescence intensity decreased as the concentration of oleic acid in the medium increased (Fig. 4c). The repression ratio of the acyl-CoA biosensor was lower than that of the malonyl-CoA biosensor, and a maximum of 42.8% repression was achieved in the presence of the saturating concentration of oleic acid.

**Fig. 5:**
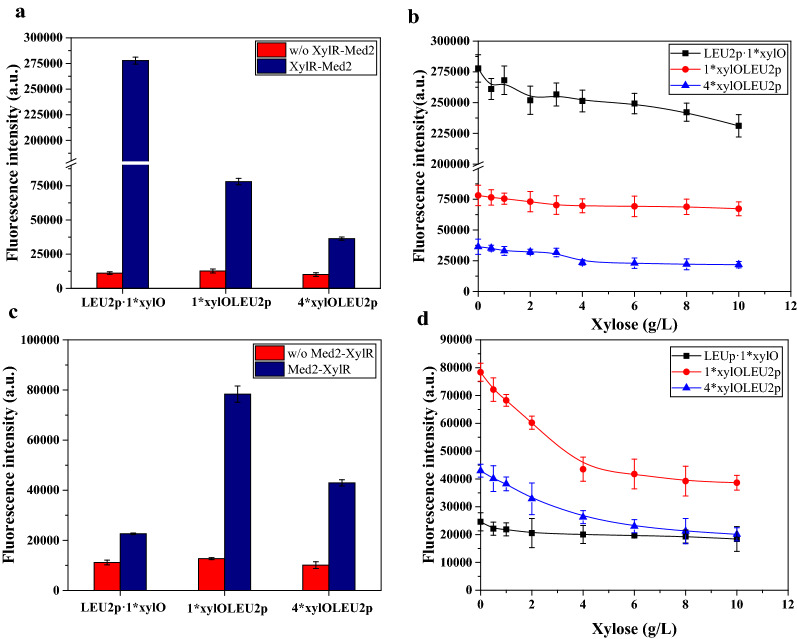
Testing of selected designs to construct xylose repressive biosensors. **a** Characterization of the transcriptional activity of strain containing “LEU2p·1*xylO”, “1*xylOLEU2p” and “4*xylOLEU2p” with or without XylR-Med2. **b** Dose–response curves of the strain containing “LEU2p·1*xylO”, “1*xylOLEU2p” and “4*xylOLEU2p” with XylR-Med2 in the presence of xylose. **c** Characterization of the transcriptional activity of the strain containing “LEU2p·1*xylO”, “1*xylOLEU2p” and “4*xylOLEU2p” with or without Med2-XylR. **d** Dose–response curves of the strain containing “LEU2p·1*xylO”, “1*xylOLEU2p” and “4*xylOLEU2p” with Med2-XylR in the presence of xylose. Minimal medium containing 2% maltose was used as the carbon source in these tests. The data are presented as the means ± standard deviations from three independent experiments

Xylose-responsive XylRs are a class of prokaryotic transcriptional repressors that contain an N-terminal DNA-binding domain and a C-terminal xylose kinase-like domain. Similar to the acyl-CoA biosensor design, *fapO* was replaced with the XylR-binding sequence *xylO* and three promoters were constructed. Because Med2 showed the best transcriptional activation in both the malonyl-CoA and acyl-CoA design, we fused XylR to Med2. Consistent with the malonyl-CoA and acyl-CoA biosensors, the strain containing Leu2p·1*xylO showed the best transcriptional activation (Fig. 5a). However, when we added different concentrations of xylose to the medium, the repressive effect was not obvious when glucose was used as the carbon source (Figure S1a). To avoid possible xylose transport by glucose, glucose was replaced with maltose as the carbon source. The repressive effect was still not obvious and the fluorescence intensity only decreased by 16.7% in the Leu2p·1*xylO containing strain (Fig. 5b).

We suspected that the fusion of Med2 affected the ligand binding of XylR. Therefore, we placed Med2 at the N-terminus of the XylR protein. Changing the position of Med2 and XylR improved the repression ratio and the sensitivity of the biosensor to xylose. Interestingly, switching the positions of XylR and Med2 greatly reduced the transcriptional activation efficiencies of the LEU2p·1*xylO design, whereas the transcriptional activation efficiency of the 1*xylO·LEU2p and 4*xylO·Leu2p designs remain unchanged (Fig. 5c). In the presence of xylose, the repression ratios of the 1*xylO·LEU2p and 4*xylO·LEU2p designs reached 50.1% and 49.6%, respectively (Fig. 5d). Using glucose or maltose as the carbon source did not affect the repression ratio (Additional file 1: Figure S1b), indicating the inhibition of xylose transport by glucose is not the reason of low repression. The above result demonstrates that the spatial positions of TF and the fusion activator are also factor that affects the ligand binding affinity.

## Discussion

In this study, we constructed biosensors that allow the repressive regulation of gene expression in response to metabolite levels in *S. cerevisiae*. The dynamic range of the biosensor was optimized by fusing different ADs, comparing promoters, and evaluating the effects of the operator numbers and positions. Among these factors, we found that the transcriptional activity of the activator is a key factor affecting the dynamic range of the biosensor. Previously, the biosensors for activational regulation were constructed by the fusion of TF with AD in eukaryotic cells [16, 34–36]. Several activators, such as VP16, B42 and Gal4, were used to construct biosensors. However, no study has compared the transcriptional activity of different activators in yeast. When we compared different activators, we found that Med2 is a very potent activator, and its transcriptional activity is even better than the strong tripartite activator VPR. Med2 is a subunit of the RNA polymerase II mediator complex, it is reported that yeast mediator subunits such as Med2 and Gal11 can activate the transcription when fusing with a DNA binding domain [37]. Among different subunits of the mediator such as Gal11, Med2, Srb10 and Srb7, Med2 showed the best activation efficiency [28]. However, the reason of its high activation activity has not been analyzed.

The position of the operator is another factor affecting the dynamic range of the biosensor. Recently, Ambri et al. investigated the impact of the TF-binding site, at single-nucleotide resolution, and demonstrated that the operator position in the promoter is one of the key factors in successful biosensor engineering, which is consistent with our findings [9]. In our study, we found that the insertion of the operator 51 bp upstream of the core element of the promoter had best activation effect. Wen et al. tested the location of *fapO* in the region of 0-86 bp upstream of core promoter of *AOX1*, and found that 52 bp upstream was the best site for transcription activation/deactivation in *Komagataella phaffii* [38]. Interestingly, we found that the operator number is not a key factor affecting the dynamic range of the biosensor. Perhaps when a strong activator is fused with TF, one operator is efficient enough to recruit the TF-AD to activate transcription. More operators do not further increase transcription, but may sterically hinder TF-AD and weaken the activation of transcription.

Although systematic engineering allowed us to construct a malonyl-CoA sensor with a high repression ratio, the repression ratios of the acyl-CoA and xylose sensors were still relatively low. Compared with the basic expression level without TF-AD, high expression was observed in these two biosensor designs when saturated concentrations of the ligand were added. It seems that TF-AD cannot be dissociated from the operator completely to deactivate the transcription, even when saturation concentrations of the ligand are added. Improving the ability of the ligand to dissociate TF from the operator is necessary to overcome this problem. Because the allosteric regulation of TFs relies on complex interdomain interactions, rational engineering may not satisfy the requirements of biosensor design. We envision that the directed evolution of TFs can be exploited in future studies to improve the repression ratios and other characteristics of biosensors.

## Conclusions

In conclusion, we successfully constructed biosensors that allowed the repressive regulation of gene expression in response to metabolite levels in *S. cerevisiae*. Using a systematic engineering approach, we have provided a framework from which new biosensors can be designed by the simple fusion of a prokaryotic transcriptional repressor with Med2 and by swapping the operator sequences in the *LEU2* promoter. Many previous studies have developed biosensors to activate gene expression in eukaryotes using prokaryotic transcriptional repressors [3, 18, 20]. Combining these with the repressive biosensor design developed here, it is now possible to construct sensors with opposing transcriptional activities in yeast. These biosensors can be applied to construct metabolic oscillator, down-regulate the byproduct synthesis pathway or screen the high metabolite producers in high-throughput. Our study will facilitate the dynamic regulation of gene expression and the development of microbial cell factories for the production of various compounds in yeast.

## Methods

### Media

YPD medium containing 20 g/L peptone and 10 g/L yeast extract, supplemented with 2% glucose, was used to culture competent yeast cells. Recombinant strains were grown in synthetic complete (SC) dropout media. SC-TRP and SC-TRP-LEU were used for yeast transformation and recombinant strain culture. They contained 1.7 g/L yeast nitrogen base (BBI Life Science Corporation, China), 5 g/L ammonium sulfate, synthetic complete drop-out medium without tryptophan and/or leucine, and 20 g/L glucose. When necessary, 400 mg/L HygB (KSE Scientific, Durham, NC) was added to the growth medium.

*Escherichia coli* strain DH5α was used to maintain and amplify plasmids, and recombinant strains were grown at 37 °C in Luria–Bertani (LB) broth (5 g/L yeast extract, 10 g/L tryptone, and 10 g/L NaCl) supplemented with 100 mg/L ampicillin.

### Plasmid and strain construction

All primer sequences and plasmids used in this study are listed in Additional file 1: Tables S1 and S2, respectively. Plasmid ligation methods included the use of restriction digestion/T4 ligase ligation and Gibson Assembly. Rhanta Max Super-Fidelity DNA Polymerase was purchased from Vazyme Biotech (Nanjing, China). Restriction enzymes and T4 ligase were purchased from Thermo Scientific (Waltham, MA).

A codon-optimized *FapR* gene from *B. subtilis*, the *FadR* gene from *E. coli*, *XylR* genes from *S. xylosus*, the *VP16* gene from herpes simplex virus, and the tripartite activator VP64-p65-Rta gene were synthesized by GenScript (Nanjing, China). The open reading frames of Med2 and Gal4 were PCR-amplified from *S. cerevisiae* genomic DNA. The synthetic linker used to connect FapR, FadR, or XylR to the Med2 mediator was GSGSGSGS. The TFs and activators were fused with overlap extension PCR and digested with *EcoR*V and *Sac*I. The fusion products FapR-ADs, FadR-Med2, XylR-Med2, and Med2-XylR were ligated into the predigested 2μ plasmid pYX242-WS under the control of the *TEF1* promoter and *PGK1* terminator, yielding the plasmids pYFapR-G, pYFapR-M, pYFapR-MG, pYFapR-V, pYFapR-VPR, pYFadR-M, pYXylR-M, and pYM-XylR.

The operators were inserted into the *LEU2* promoter (250 bp), which contains the 125-bp identified core sequence and the 125-bp upstream activation sequence. One FapR-binding site (17 bp of the *fapO* operator: TTAGTATCAGGTACTAA) was inserted into the upstream region of *LEU2p*, 51 bp upstream from the core promoter, to generate the synthetic promoter LEU2p·1*fapO. Using similar design principles, one FadR-binding site (17 bp of the *fadO* operator: ATCTGGTACGACCAGAT) and one XylR-binding site (29 bp of the *xylO* operator: AGTTAGTTTGTTTATTAAATTAACCAACT) were used to replace the *fapO* sequence to generate the synthetic promoters LEU2p·1*fadO and LEU2p·1*xylO, respectively. Two *fapO* sites with an interval of 8 bp were inserted into the same position to generate the synthetic promoter LEU2p·2*fapO. One *fapO* site was inserted 21 bp upstream of the *LEU2p* upstream region to generate the synthetic promoter 1*fapO·LEU2p. Two *fapO* sites were inserted 21 bp upstream of the *LEU2p* upstream region to generate the synthetic promoter 2*fapO·LEU2p. Four *fapO*, *fadO*, or *xylO* sites were inserted 21 bp upstream of the *LEU2p* upstream region to generate synthetic promoters 4*fapO·LEU2p, 4*fadO·LEU2p, or 4*xylO·LEU2p, respectively. The *LEU2* core promoter of the synthetic promoter LEU2p·1*fapO was replaced with a 59-bp synthetic minimum core promoter to generate the synthetic promoter LEU2p·1*fapOCore1p. Four *fapO* sites were inserted, at intervals of 8 bp, 51 bp upstream from the synthetic minimum core promoter to generate the synthetic promoter 4*fapOCore1p. Four *fapO* sites were inserted 21 bp upstream from the *LEU2* core promoter to generate the synthetic promoter 4*fapO·LEU2cp. Each designed synthetic promoter was fused to the reporter gene yeGFP (the yeast enhanced green fluorescent protein [EGFP]-encoding gene) with overlap extension PCR. The fragment was then digested with *Sac*I and *Sbf*I and inserted into the pre-digested yeast integrative plasmid pRS304 to generate pRS304-01-pRS304-14. Transformation of these plasmids produced strains Qse01-Qse14, respectively. All the strains used in this study are listed in Additional file 1: Table S3.

*Saccharomyces cerevisiae* CEN.PK2-1C (MATa; ura3-52; trp1-289; leu2-3,112; his3Δ1; MAL2-8C; SUC2) was used as the host for all homologous-recombination-based cloning, sensor construction, and optimization. All *S. cerevisiae* transformations were performed with the lithium acetate method [39].

### Fluorescence intensity measurement

Fluorescence was measured with a 1420 Multilabel Counter (Victor3™ V, PerkinElmer, USA). The excitation and emission wavelengths for GFP were 485 ± 20 and 585 ± 20 nm, respectively. Cell density was measured at 600 nm (Eppendorf BioPhotometer, Germany). The fluorescence intensity (a. u.) was determined relative to the cell density. The strains were grown overnight at 30 °C with agitation at 200 rpm. For the malonyl-CoA response assays, an overnight culture was collected and used to inoculate 4 mL of fresh SC medium, with an initial optical density at a wavelength of 600 nm (OD_600_) of 0.2. Cerulenin was added at a final concentration of 0, 5, 10, 15, or 20 μM. After culture at 30 °C for approximately 12 h, the fluorescence intensity was measured. Similarly, for the acyl-CoA response assay, 0, 0.1, 0.2, 0.5, 1, or 2 mM oleic acid was added. For the xylose response assay, 2% glucose or maltose was used as the carbon source and 0, 0.5, 1, 2, 4, 6, 8, or 10 g/L xylose was added. Three independent replicates were cultivated and the fluorescence was measured.

## Supplementary information

**Additional file 1: Table S1**. Oligo-nucleotide primer sequences used to construct plasmids in this study. **Table S2**. Plasmids used in this study. **Table S3**. Strains used in this study. **Figure S1**: Characterization of xylose repression sensor in a minimal medium containing 2% glucose as the carbon source.

## Data Availability

All data generated or analyzed during this study are included in this published article and its supplementary information files.

## Abbreviations

TFs: Transcription factors
GFP: Green fluorescent protein
AD: Activation domain

## References

[1] Michener JK, Thodey K, Liang JC, Smolke CD (2012). Applications of genetically-encoded biosensors for the construction and control of biosynthetic pathways. Metab Eng.

[2] Walker RS, Pretorius IS (2018). Applications of yeast synthetic biology geared towards the production of biopharmaceuticals. Genes.

[3] David F, Nielsen J, Siewers V (2016). Flux control at the malonyl-CoA node through hierarchical dynamic pathway regulation in *Saccharomyces cerevisiae*. ACS Synth Biol.

[4] Li S, Si T, Wang M, Zhao H (2015). Development of a synthetic Malonyl-CoA sensor in *Saccharomyces cerevisiae* for intracellular metabolite monitoring and genetic screening. ACS Synth Biol.

[5] Ferreira R, Skrekas C, Hedin A, Sanchez BJ, Siewers V, Nielsen J, David F (2019). Model-assisted fine-tuning of central carbon metabolism in yeast through dCas9-based regulation. ACS Synth Biol.

[6] Chen X, Yang X, Shen Y, Hou J, Bao X (2018). Screening phosphorylation site mutations in yeast acetyl-CoA carboxylase using malonyl-CoA sensor to improve malonyl-CoA-derived product. Front Microbiol.

[7] Qiu C, Zhai H, Hou J (2019). Biosensors design in yeast and applications in metabolic engineering. FEMS Yeast Research..

[8] Dabirian Y, Li X, Chen Y, David F, Nielsen J, Siewers V (2019). Expanding the dynamic range of a transcription factor-based biosensor in *Saccharomyces cerevisiae*. ACS Synth Biol.

[9] Ambri F, D’Ambrosio V, Di Blasi R, Maury J, Jacobsen SAB, McCloskey D, Jensen MK, Keasling JD (2020). High-resolution scanning of optimal biosensor reporter promoters in yeast. ACS Synth Biol.

[10] Skjoedt ML, Snoek T, Kildegaard KR, Arsovska D, Eichenberger M, Goedecke TJ, Rajkumar AS, Zhang J, Kristensen M, Lehka BJ (2016). Engineering prokaryotic transcriptional activators as metabolite biosensors in yeast. Nat Chem Biol.

[11] D’Ambrosio V, Jensen MK: Lighting up yeast cell factories by transcription factor-based biosensors. *FEMS Yeast Res* 2017, 17. 10.1093/femsyr/fox076 PMC5812511 28961766

[12] Mahr R, Frunzke J (2016). Transcription factor-based biosensors in biotechnology: current state and future prospects. Appl Microbiol Biotechnol.

[13] Xu P, Li L, Zhang F, Stephanopoulos G, Koffas M (2014). Improving fatty acids production by engineering dynamic pathway regulation and metabolic control. Proc Natl Acad Sci U S A.

[14] Solomon KV, Sanders TM, Prather KL (2012). A dynamic metabolite valve for the control of central carbon metabolism. Metab Eng.

[15] Lee SW, Oh MK (2015). A synthetic suicide riboswitch for the high-throughput screening of metabolite production in *Saccharomyces cerevisiae*. Metab Eng.

[16] Belli G, Gari E, Piedrafita L, Aldea M, Herrero E (1998). An activator/repressor dual system allows tight tetracycline-regulated gene expression in budding yeast. Nucleic Acids Res.

[17] Snoek T, Chaberski EK, Ambri F, Kol S, Bjorn SP, Pang B, Barajas JF, Welner DH, Jensen MK, Keasling JD (2020). Evolution-guided engineering of small-molecule biosensors. Nucleic Acids Res.

[18] Teo WS, Chang MW (2015). Bacterial XylRs and synthetic promoters function as genetically encoded xylose biosensors in *Saccharomyces cerevisiae*. Biotechnol J.

[19] Wang M, Li S, Zhao H (2016). Design and engineering of intracellular-metabolite-sensing/regulation gene circuits in *Saccharomyces cerevisiae*. Biotechnol Bioeng.

[20] Teo WS, Hee KS, Chang MW (2013). Bacterial FadR and synthetic promoters function as modular fatty acid sensor- regulators in *Saccharomyces cerevisiae*. Eng Life Sci.

[21] Dabirian Y, Goncalves Teixeira P, Nielsen J, Siewers V, David F (2019). FadR-based biosensor-assisted screening for genes enhancing fatty Acyl-CoA pools in *Saccharomyces cerevisiae*. ACS Synth Biol.

[22] Zhang G, Liao L, Lin Y, Yang M, Xiao X, Nie C (2013). Determination of fructose 1,6-bisphosphate using a double-receptor sandwich type fluorescence sensing method based on uranyl-salophen complexes. Anal Chim Acta.

[23] Ptashne M (1988). How eukaryotic transcriptional activators work. Nature.

[24] Teo WS, Chang MW (2014). Development and characterization of AND-gate dynamic controllers with a modular synthetic *GAL1* core promoter in *Saccharomyces cerevisiae*. Biotechnol Bioeng.

[25] Schujman GE, Guerin M, Buschiazzo A, Schaeffer F, Llarrull LI, Reh G, Vila AJ, Alzari PM, de Mendoza D (2006). Structural basis of lipid biosynthesis regulation in Gram-positive bacteria. EMBO J.

[26] Tu H, Casadaban MJ (1990). The upstream activating sequence for l-leucine gene regulation in *Saccharomyces cerevisiae*. Nucleic Acids Res.

[27] Sadowski I, Ma J, Triezenberg S, Ptashne M (1988). GAL4-VP16 is an unusually potent transcriptional activator. Nature.

[28] Balciunas D, Hallberg M, Bjorklund S, Ronne H (2003). Functional interactions within yeast mediator and evidence of differential subunit modifications. J Biol Chem.

[29] Lian J, HamediRad M, Hu S, Zhao H (2017). Combinatorial metabolic engineering using an orthogonal tri-functional CRISPR system. Nat Commun.

[30] Ohno H, Ohno T, Awaya J, Omura S (1975). Inhibition of 6-methylsalicyclic acid synthesis by the antibiotic cerulenin. J Biochem.

[31] Redden H, Alper HS (2015). The development and characterization of synthetic minimal yeast promoters. Nat Commun.

[32] Blazeck J, Garg R, Reed B, Alper HS (2012). Controlling promoter strength and regulation in *Saccharomyces cerevisiae* using synthetic hybrid promoters. Biotechnol Bioeng.

[33] Henry MF, Cronan JE (1992). A new mechanism of transcriptional regulation: release of an activator triggered by small molecule binding. Cell.

[34] Gossen M, Bujard H (1992). Tight control of gene expression in mammalian cells by tetracycline-responsive promoters. Proc Natl Acad Sci U S A.

[35] Moser F, Horwitz A, Chen J, Lim W, Voigt CA (2013). Genetic sensor for strong methylating compounds. ACS Synth Biol.

[36] Umeyama T, Okada S, Ito T (2013). Synthetic gene circuit-mediated monitoring of endogenous metabolites: identification of *GAL11* as a novel multicopy enhancer of s-adenosylmethionine level in yeast. ACS Synth Biol.

[37] Wang X, Muratani M, Tansey WP, Ptashne M (2010). Proteolytic instability and the action of nonclassical transcriptional activators. Curr Biol.

[38] Wen J, Tian L, Xu M, Zhou X, Zhang Y, Cai M (2020). A synthetic Malonyl-CoA metabolic oscillator in *Komagataella phaffii*. ACS synthetic biology.

[39] Gietz RD, Woods RA (2002). Transformation of yeast by lithium acetate/single-stranded carrier DNA/polyethylene glycol method. Methods Enzymol.

