# Supplementary material for: Engineering transcription factor-based biosensors for repressive regulation through transcriptional deactivation design in Saccharomyces cerevisiae
Source: Microb Cell Fact. 2020 Jul 20;19:146. doi: 10.1186/s12934-020-01405-1 (PMC7372789; doi:10.1186/s12934-020-01405-1)
Supplement: Supplementary file 1 — Additional file 1: Table S1. Oligo-nucleotide primer sequences used to construct plasmids in this study. Table S2. Plasmids used in this study. Table S3. Strains used in this study. Figure S1: Characterization of xylose repression sensor in a minimal medium containing 2% glucose as the carbon source. [file 12934_2020_1405_MOESM1_ESM.doc]

**Engineering transcription factor-based biosensors for repressive regulation through transcriptional deactivation design in *Saccharomyces cerevisiae***

Chenxi Qiu^1,#^, Xiaoxu Chen^1,#, †^, Reheman Rexida^1^, Yu Shen^1^, Qingsheng Qi^1^, Xiaoming Bao^1, 2^, Jin Hou^1,*^

^1^ State Key Laboratory of Microbial Technology, Shandong University, Qingdao, 266237, P. R. China

^2^ State Key Laboratory of Biobased Material and Green Papermaking, School of Bioengineering, Qi Lu University of Technology, Jinan, 250353, P. R. China.

^†^Present address: State Key Laboratory of Natural and Biomimetic Drugs, School of Pharmaceutical Sciences, Peking University, Beijing, 100191, P. R. China

^#^To be considered joint first authors.

*Corresponding author: Prof. Jin Hou, State Key Laboratory of Microbial Technology, Shandong University, Binhai Road 72, Qingdao, Shandong, 266237, P. R. China;

Tel: +86 531 8836 5827; Fax: +86531 8836 5826.

email: houjin@sdu.edu.cn

**SUPPORTING INFORMATION**

**Table S1**. Oligo-nucleotide primer sequences used to construct plasmids in this study.

| Primer | Sequence (5’-3’) | Description |
| --- | --- | --- |
| LEU2p-F | CGAGCTCGCATATACCTTTTTCAACTGAAAAATTG | For pRS304-01, pRS304-02, pRS304-06, pRS304-09, pRS304-12 and |
| LEU2-GFP-R | GAATAATTCTTCACCTTTAGACATCTCGAGTAGAATGGTATATCCTTGAAATATATATATATATATTG | pRSLEU2p-GFP  construction |
| GFP-LEU2-F | ATATATTTCAAGGATATACCATTCTACTCGAGATGTCTAAAGGTGAAGAATTATTCAC |  |
| GFP-R | GTCCCCTGCAGGTTATTTGTACAATTCATCCATACC |  |
| fapO- LEU2p-F | CGCGAGCTCGAGTTAGTTTGTTTATTAAATTAACCAACTTAATTTCAGAG | For pRS304-03 construction |
| 2fapO-LEU2p-F | CGCGAGCTCGAGTTAGTTAGTACCTGATACTAATTTTGTCTTTAG | For pRS304-04, pRS304-05, pRS304-12  and pRS304-14 construction |
| fadO-LEU2p-F | CGCGAGCTCGATCTGGTACGACCAGATTAATTTC | For pRS304-07  construction |
| 4fadO-LEU2p-F | CGCGAGCTCGATCTGGTACGACCAGATTTTG | For pRS304-08  construction |
| xylO-LEU2p-F | CGCGAGCTCGAGTTAGTTTGTTTATTAAATTAACCAAC | For pRS304-10  and pRS11 construction |
| GFP-Core1p-F | CCGGAAGCATCGAAAAAATCTAGACCTCGAGATGTCTAAAGGTGAAGAATTATTCAC | For pRS304-12 and pRS304-13  construction |
| Core1-GFP-R | CAGTGAATAATTCTTCACCTTTAGACATCTCGAGGTCTAGATTTTTTCGATGCTTCC |
| FapR-F | GCATGATATCTCATGAGAAGAAATAAAAGAGAAAGGCAAG | For pYFapR-M  construction |
| FapR-Med2-R | GATCCTGATCCAGAACCAGAACCAGAATGTTTAGATCTATACATATCGAATCTACC |
| Med2-FapR-F | GTTCTGGTTCTGGATCAGGATCAATGGTAGTACAAAATAGCCCAGTTT |
| Med2-R | CGAGCTCGCTATATATTGAAGCCGCTGAGGTC |
| Gal4-FapR-F | CTGGTTCTGGTTCTGGATCAGGATCAGCCAATTTTAATCAAAGTGGG | For pYFapR-G  construction |
| FapR-Gal4-R | GATCCTGATCCAGAACCAGAACCAGAATGTTTAGATCTATACATATCGAATCTACC |
| Gal4-R | CGAGCTCGCATCCTCTTTTTTTGGGTTTGGTGG |
| VP16-FapR-F | CTGGTTCTGGTTCTGGATCAGGATCAATGGAAGAAGAATCTTCCACTG | For pYFapR-V  construction |
| FapR-VP16-R | GATCCTGATCCAGAACCAGAACCAGAATGTTTAGATCTATACATATCGAATCTAC |
| VP16-R | CGAGCTCGCTTAGGGCATAGGTATATCAAGG |
| Med2-Gal4-R | GGCTGATCCTGATCCAGAACCAGAACCTATATTGAAGCCGCTGAGGTC | For pYFapR-MG  construction |
| Gal4-Med2-F | GAATTAGACCTCAGCGGCTTCAATATAGGTTCTGGTTCTGGATCAGG |
| VP64-FapR-F | CGATATGTATAGATCTAAACATTCTGGTTCTGGTTCTGGATCAGG | For pYFapR-VPR  construction |
| FapR-VP64-F | CTCTGATCCTGATCCAGAACCAGAACCAGAATGTTTAGATCTATACATATCGAATC |
| TRA-R | ATGCGATATCATGCTCAAAACAGAGATGTGTCGAAGATGG |
| FadR-F | GCATGATATCTCATGGTCATTAAGGCGCAAAG | For pYFadR-M  construction |
| Med2-FadR-F | CCGGGTGATTTAGCCATTCAGGGCGAGGTTCTGGTTCTGGATCAGG |
| FadR-Med2-R | CCATTGATCCTGATCCAGAACCAGAACCTCGCCCCTGAATGGCTAAATC |
| VP64-FadR-F | GGCGAGGTTCTGGTTCTGGATCAGGATCAGAGGCCAGCGGTTCCGG | For pYFadR-VPR  construction |
| FadR-VP64-R | CAGCCCGTCCGGAACCGCTGGCCTCTGATCCTGATCCAGAACCAGAAC |
| XylR-F | GCATGATATCTCATGGAAAATAATTTTATAGTAAATGAAAATG | For pYXylR-M  construction |
| Med2-XylR-F | TATAAATAACATTCAAATGAATATTAAAGGTTCTGGTTCTGGATCAG |
| XylR-Med2-R | CTGATCCAGAACCAGAACCTTTAATATTCATTTGAATGTTATTTATATTTAATGTTTTTTG |
| Med2-F | GCATGATATCTCATGGTAGTACAAAATAGCCCAG | For pYM-XylR  construction |
| XylR-Med2-F | GAATTAGACCTCAGCGGCTTCAATATAGGTTCTGGTTCTGGATCAGG |
| Med2-XylR-R | CATTGATCCTGATCCAGAACCAGAACCTATATTGAAGCCGCTGAGGTC |

**Table S2**. Plasmids used in this study

| Plasmid | Description | Source |
| --- | --- | --- |
| pRS304 | Yeast integration plasmid with *TRP1* maker | Lab store |
| pIYC04 | 2µm *ori*, *HIS3*, *TEF1p*-*ADH1t*, *PGK1p-CYC1t* | [1] |
| pJFE1 | *CEN4*, *URA3*,*TEF1p-PGK1t* | Lab store |
| pYX242-WS | 2µm *ori*, *LEU2*, *TEF1p-PGK1t* | Lab store |
| pYFapR-M | pYX242-*TEF1p*-*FapR-Med2-PGK1t* | This study |
| pYFapR-G | pYX242-*TEF1p-FapR-Gal4-PGK1t* | This study |
| pYFapR-V | pYX242-*TEF1p*-*FapR-VP16-PGK1t* | This study |
| pYFapR-MG | pYX242-*TEF1p*-*FapR-Med2-Gal4-PGK1t* | This study |
| pYFapR-VPR | pYX242-*TEF1p-FapR-VP64-p65-Rta-PGK1t* | This study |
| pYFadR-M | pYX242-*TEF1p-FadR-Med2-PGK1t* | This study |
| pYXylR-M | pYX242-*TEF1p*-*xylR-Med2-PGK1t* | This study |
| pYM-XylR | pYX242-*TEF1p-Med2*-*xylR-PGK1t* | This study |
| pRS304-01 | pRS304*-LEU2p·1*fapO-GFP-PGK1t* | This study |
| pRS304-02 | pRS304*- LEU2p·2*fapO-GFP-PGK1t* | This study |
| pRS304-03 | pRS304-*1*fapO·LEU2p-GFP-PGK1t* | This study |
| pRS304-04 | pRS304*-2*fapO·LEU2p-GFP-PGK1t* | This study |
| pRS304-05 | pRS304*-4*fapO·LEU2p-GFP-PGK1t* | This study |
| pRS304-06 | pRS304*-LEU2p·1*fadO-GFP-PGK1t* | This study |
| pRS304-07 | pRS304-*1*fadO·LEU2p-GFP-PGK1t* | This study |
| pRS304-08 | pRS304-*4*fapO·LEU2p-GFP-PGK1t* | This study |
| pRS304-09 | pRS304*-LEU2p·1*xylO-GFP-PGK1t* | This study |
| pRS304-10 | pRS304-*1*xylO·LEU2p-GFP-PGK1t* | This study |
| pRS304-11 | pRS304-*4*xylO·LEU2p-GFP-PGK1t* | This study |
| pRS304-12 | pRS304-*LEU2p·1*fapOCore1p-GFP-PGK1t* | This study |
| pRS304-13 | pRS304-*4*fapOCore1p-GFP-PGK1t* | This study |
| pRS304-14 | pRS304-*4*fapO·LEU2cp-GFP-PGK1t* | This study |

**Table S3**. Strains used in this study

| Strain | Genotype | Source |
| --- | --- | --- |
| CEN.PK2-1C | *MATa; ura3-52; trp1-289;leu2-3,112; his3Δ1; MAL2-8C; SUC2* | This study |
| QCse01 | CEN.PK2-1C; *trp1*::*LEU2p·1*fapO-GFP-PGK1t* | This study |
| QCse02 | CEN.PK2-1C; *trp1::LEU2p·2*fapO-GFP-PGK1t* | This study |
| QCse03 | CEN.PK2-1C; *trp1::1*fapO·LEU2p-GFP-PGK1t* | This study |
| QCse04 | CEN.PK2-1C; *trp1::2*fapO·LEU2p-GFP-PGK1t* | This study |
| QCse05 | CEN.PK2-1C; *trp1::4*fapO·LEU2p-GFP-PGK1t* | This study |
| QCse06 | CEN.PK2-1C; *trp1::LEU2p·1*fadO-GFP-PGK1t* | This study |
| QCse07 | CEN.PK2-1C; *trp1::1*fadO·LEU2p-GFP-PGK1t* | This study |
| QCse08 | CEN.PK2-1C; *trp1::4*fadO·LEU2p-GFP*-*PGK1t* | This study |
| QCse09 | CEN.PK2-1C; *trp1::LEU2p·1*xylO-GFP-PGK1t* | This study |
| QCse10 | CEN.PK2-1C; *trp1::1*xylO·LEU2p-GFP-PGK1t* | This study |
| QCse11 | CEN.PK2-1C; *trp1::4*xylO·LEU2p-GFP-PGK1t* | This study |
| QCse12 | CEN.PK2-1C; *trp1::LEU2p·1*fapOCore1p-GFP-PGK1t* | This study |
| QCse13 | CEN.PK2-1C; *trp1::4*fapOCore1p-GFP-PGK1t* | This study |
| QCse14 | CEN.PK2-1C; *trp1::4*fapO·LEU2cp-GFP-PGK1t* | This study |
| QCse15 | CEN.PK2-1C; *trp1*::*LEU2p·1*fapO-GFP-PGK1t*, pYFapR-M | This study |
| QCse16 | CEN.PK2-1C; *trp1::LEU2p·2*fapO-GFP-PGK1t*, pYFapR-M | This study |
| QCse17 | CEN.PK2-1C; *trp1::1*fapO·LEU2p-GFP-PGK1t*, pYFapR-M | This study |
| QCse18 | CEN.PK2-1C; *trp1::2*fapO·LEU2p-GFP-PGK1t*, pYFapR-M | This study |
| QCse19 | CEN.PK2-1C; *trp1::4*fapO·LEU2p-GFP-PGK1t*, pYFapR-M | This study |
| QCse20 | CEN.PK2-1C; *trp1::4*fapO·LEU2p-GFP-PGK1t*, pYFapR-G | This study |
| QCse21 | CEN.PK2-1C; *trp1::4*fapO·LEU2p-GFP-PGK1t*, pYFapR-V | This study |
| QCse22 | CEN.PK2-1C; *trp1::4*fapO·LEU2p-GFP-PGK1t*, pYFapR-MG | This study |
| QCse23 | CEN.PK2-1C; *trp1::4*fapO·LEU2p-GFP-PGK1t*, pYFapR-VPR | This study |
| QCse24 | CEN.PK2-1C; *trp1::1*fadO·LEU2p-GFP-PGK1t*, pYFadR-M | This study |
| QCse25 | CEN.PK2-1C; *trp1::1*fadO·LEU2p-GFP-PGK1t*, pYFadR-M | This study |
| QCse26 | CEN.PK2-1C; *trp1::4*fadO·LEU2p-GFP-PGK1t*, pYFadR-M | This study |
| QCse27 | CEN.PK2-1C; *trp1::LEU2p·1*xylO-GFP-PGK1t*, pYXylR-M | This study |
| QCse28 | CEN.PK2-1C; *trp1::1*xylO·LEU2p-GFP-PGK1t*, pYXylR-M | This study |
| QCse29 | CEN.PK2-1C; *trp1::4*xylO·LEU2p-GFP-PGK1t*, pYXylR-M | This study |
| QCse30 | CEN.PK2-1C; *trp1::4* LEU2p·1*xylO -GFP-PGK1t*,  pYM-XylR | This study |
| QCse31 | CEN.PK2-1C; *trp1::1*xylO·LEU2p-GFP-PGK1t*,  pYM-XylR | This study |
| QCse32 | CEN.PK2-1C; *trp1::4*xylO·LEU2p-GFP-PGK1t*,  pYM-XylR | This study |
| QCse33 | CEN.PK2-1C; *trp1::LEU2p·1*fapOCore1p-GFP-PGK1t*, pYFapR-M | This study |
| QCse34 | CEN.PK2-1C; *trp1::4*fapOCore1p-GFP-PGK1t*, pYFapR-M | This study |
| QCse35 | CEN.PK2-1C; *trp1::4*fapO·LEU2cp-GFP-PGK1t*, pYFapR-M | This study |

**Sequences.** Synthetic gene sequences.

**Synthetic promoter sequence of *LEU2p·1*fapO***

GCATATACCTTTTTCAACTGAAAAATTGGGAGAAAAAGGAAAGGTGAGAGCGCCGGAACCGGCTTTTCATATAGTTAGTACCTGATACTAAAATAGAGAAGCGTTCATGACTAAATGCTTGCATCACAATACTTGAAGTTGACAATATTATTTAAGGACCTATTGTTTTTTCCAATAGGTGGTTAGCAATCGTCTTACTTTCTAACTTTTCTTACCTTTTACATTTCAGCAATATATATATATATATTTCAAGGATATACCATTCTA

**Synthetic promoter sequence of *LEU2p·2*fapO***

GCATATACCTTTTTCAACTGAAAAATTGGGAGAAAAAGGAAAGGTGAGAGCGCCGGAACCGGCTTTTCATATAGTTAGTACCTGATACTAAttttgtctTTAGTACCTGATACTAAAATAGAGAAGCGTTCATGACTAAATGCTTGCATCACAATACTTGAAGTTGACAATATTATTTAAGGACCTATTGTTTTTTCCAATAGGTGGTTAGCAATCGTCTTACTTTCTAACTTTTCTTACCTTTTACATTTCAGCAATATATATATATATATTTCAAGGATATACCATTCTA

**Synthetic promoter sequence of *1*fapO·LEU2p***

TTAGTACCTGATACTAATAATTTCAGAGGTCGCCTGACGCATATACCTTTTTCAACTGAAAAATTGGGAGAAAAAGGAAAGGTGAGAGCGCCGGAACCGGCTTTTCATATAGAATAGAGAAGCGTTCATGACTAAATGCTTGCATCACAATACTTGAAGTTGACAATATTATTTAAGGACCTATTGTTTTTTCCAATAGGTGGTTAGCAATCGTCTTACTTTCTAACTTTTCTTACCTTTTACATTTCAGCAATATATATATATATATTTCAAGGATATACCATTCTA

**Synthetic promoter sequence of *2*fapO·LEU2p***

TTAGTACCTGATACTAAttttgtctTTAGTACCTGATACTAATAATTTCAGAGGTCGCCTGACGCATATACCTTTTTCAACTGAAAAATTGGGAGAAAAAGGAAAGGTGAGAGCGCCGGAACCGGCTTTTCATATAGAATAGAGAAGCGTTCATGACTAAATGCTTGCATCACAATACTTGAAGTTGACAATATTATTTAAGGACCTATTGTTTTTTCCAATAGGTGGTTAGCAATCGTCTTACTTTCTAACTTTTCTTACCTTTTACATTTCAGCAATATATATATATATATTTCAAGGATATACCATTCTA

**Synthetic promoter sequence of *4*fapO·LEU2p***

TTAGTACCTGATACTAAttttgtctTTAGTACCTGATACTAAttttgtctTTAGTACCTGATACTAAttttgtctTTAGTACCTGATACTAATAATTTCAGAGGTCGCCTGACGCATATACCTTTTTCAACTGAAAAATTGGGAGAAAAAGGAAAGGTGAGAGCGCCGGAACCGGCTTTTCATATAGAATAGAGAAGCGTTCATGACTAAATGCTTGCATCACAATACTTGAAGTTGACAATATTATTTAAGGACCTATTGTTTTTTCCAATAGGTGGTTAGCAATCGTCTTACTTTCTAACTTTTCTTACCTTTTACATTTCAGCAATATATATATATATATTTCAAGGATATACCATTCTA

**Synthetic promoter sequence of *LEU2p·1*fadO***

GCATATACCTTTTTCAACTGAAAAATTGGGAGAAAAAGGAAAGGTGAGAGCGCCGGAACCGGCTTTTCATATAGAATAGAGAAGCGTTCATGACTAAATGCTTGCATCACAATACTTGAAGTTGAATCTGGTACGACCAGATAATAGAGAAGCGTTCATGACTAAATGCTTGCATCACAATACTTGAAGTTGACAATATTATTTAAGGACCTATTGTTTTTTCCAATAGGTGGTTAGCAATCGTCTTACTTTCTAACTTTTCTTACCTTTTACATTTCAGCAATATATATATATATATTTCAAGGATATACCATTCTA

**Synthetic promoter sequence of *1*fadO·LEU2p***

ATCTGGTACGACCAGATTAATTTCAGAGGTCGCCTGACGCATATACCTTTTTCAACTGAAAAATTGGGAGAAAAAGGAAAGGTGAGAGCGCCGGAACCGGCTTTTCATATAGAATAGAGAAGCGTTCATGACTAAATGCTTGCATCACAATACTTGAAGTTGACAATATTATTTAAGGACCTATTGTTTTTTCCAATAGGTGGTTAGCAATCGTCTTACTTTCTAACTTTTCTTACCTTTTACATTTCAGCAATATATATATATATATTTCAAGGATATACCATTCTA

**Synthetic promoter sequence of *4*fadO·LEU2p***

ATCTGGTACGACCAGATttttgtctATCTGGTACGACCAGATttttgtctATCTGGTACGACCAGATttttgtctATCTGGTACGACCAGATTAATTTCAGAGGTCGCCTGACGCATATACCTTTTTCAACTGAAAAATTGGGAGAAAAAGGAAAGGTGAGAGCGCCGGAACCGGCTTTTCATATAGAATAGAGAAGCGTTCATGACTAAATGCTTGCATCACAATACTTGAAGTTGACAATATTATTTAAGGACCTATTGTTTTTTCCAATAGGTGGTTAGCAATCGTCTTACTTTCTAACTTTTCTTACCTTTTACATTTCAGCAATATATATATATATATTTCAAGGATATACCATTCTA

**Synthetic promoter sequence of *LEU2p·1*xylO***

GCATATACCTTTTTCAACTGAAAAATTGGGAGAAAAAGGAAAGGTGAGAGCGCCGGAACCGGCTTTTCATATAGAATAGAGAAGCGTTCATGACTAAATGCTTGCATCACAATACTTGAAGTTGAAGTTAGTTTGTTTATTAAATTAACCAACTAATAGAGAAGCGTTCATGACTAAATGCTTGCATCACAATACTTGAAGTTGACAATATTATTTAAGGACCTATTGTTTTTTCCAATAGGTGGTTAGCAATCGTCTTACTTTCTAACTTTTCTTACCTTTTACATTTCAGCAATATATATATATATATTTCAAGGATATACCATTCTA

**Synthetic promoter sequence of *1*xylO·LEU2p***

AGTTAGTTTGTTTATTAAATTAACCAACTTAATTTCAGAGGTCGCCTGACGCATATACCTTTTTCAACTGAAAAATTGGGAGAAAAAGGAAAGGTGAGAGCGCCGGAACCGGCTTTTCATATAGAATAGAGAAGCGTTCATGACTAAATGCTTGCATCACAATACTTGAAGTTGACAATATTATTTAAGGACCTATTGTTTTTTCCAATAGGTGGTTAGCAATCGTCTTACTTTCTAACTTTTCTTACCTTTTACATTTCAGCAATATATATATATATATTTCAAGGATATACCATTCTA

**Synthetic promoter sequence of *4*xylO·LEU2p***

AGTTAGTTTGTTTATTAAATTAACCAACTttttgtctAGTTAGTTTGTTTATTAAATTAACCAACTttttgtctAGTTAGTTTGTTTATTAAATTAACCAACTttttgtctAGTTAGTTTGTTTATTAAATTAACCAACTTAATTTCAGAGGTCGCCTGACGCATATACCTTTTTCAACTGAAAAATTGGGAGAAAAAGGAAAGGTGAGAGCGCCGGAACCGGCTTTTCATATAGAATAGAGAAGCGTTCATGACTAAATGCTTGCATCACAATACTTGAAGTTGACAATATTATTTAAGGACCTATTGTTTTTTCCAATAGGTGGTTAGCAATCGTCTTACTTTCTAACTTTTCTTACCTTTTACATTTCAGCAATATATATATATATATTTCAAGGATATACCATTCTA

**Synthetic promoter sequence of *LEU2p·1*fapOCore1p***

GCATATACCTTTTTCAACTGAAAAATTGGGAGAAAAAGGAAAGGTGAGAGCGCCGGAACCGGCTTTTCATATAGTTAGTACCTGATACTAAAATAGAGAAGCGTTCATGACTAAATGCTTGCATCACAATACTTGAAGTTGATTAATTAACTTGTAATATTCTAATCAAGCTTATAAAAGAGCACTGTTGGGCGTGAGTGGAGGCGCCGGAAGCATCGAAAAAATCTAGAC

**Synthetic promoter sequence of *4*fapOCore1p***

TTAGTACCTGATACTAAttttgtctTTAGTACCTGATACTAAttttgtctTTAGTACCTGATACTAAttttgtctTTAGTACCTGATACTAATAATTTCAGAGGTCGCCTGACTTAATTAACTTGTAATATTCTAATCAAGCTTATAAAAGAGCACTGTTGGGCGTGAGTGGAGGCGCCGGAAGCATCGAAAAAATCTAGAC

**Synthetic promoter sequence of *4*fapO·LEU2cp***

TTAGTACCTGATACTAAttttgtctTTAGTACCTGATACTAAttttgtctTTAGTACCTGATACTAAttttgtctTTAGTACCTGATACTAATTAATTAACTTGTAATATTCTAATCAAGCTTCAATATTATTTAAGGACCTATTGTTTTTTCCAATAGGTGGTTAGCAATCGTCTTACTTTCTAACTTTTCTTACCTTTTACATTTCAGCAATATATATATATATATTTCAAGGATATACCATTCTA

Note: The red font indicates the UAS sequence of the *LEU2* promoter, and the green font indicates the core promoter sequence of the *LEU2* promoter. The purple font indicates the sequence of the *Core1* promoter. The yellow shaded portion indicates the sequence of *fapO*, the blue shaded portion indicates the sequence of *fadO*, and the gray shaded portion indicates the shaded portion of *xylO*.

**Condon optimized sequence of *fapR***

ATGAGAAGAAATAAAAGAGAAAGGCAAGAATTGTTGCAACAAACTATTCAAGCTACTCCATTCATTACTGATGAAGAATTAGCTGGTAAATTTGGTGTTTCTATTCAAACTATTAGATTAGATAGATTGGAATTGTCTATTCCAGAATTGAGAGAAAGAATTAAAAATGTTGCTGAAAAAACTTTGGAAGATGAAGTTAAATCTTTGTCTTTAGATGAAGTTATTGGTGAAATTATTGATTTGGAATTGGACGATCAAGCTATTTCTATTTTGGAAATTAAGCAAGAACATGTTTTTTCAAGAAATCAAATTGCTAGAGGTCACCACTTGTTCGCACAGGCTAACTCTTTGGCTGTCGCTGTCATTGACGACGAATTGGCTTTGACAGCTTCTGCTGACATTAGATTTACTAGACAGGTCAAGCAAGGTGAAAGGGTCGTTGCAAAGGCAAAGGTCACTGCTGTCGAGAAGGAGAAGGGTAGAACTGTCGTCGAGGTCAACTCTTACGTCGGTGAAGAAATTGTTTTTTCTGGTAGATTCGATATGTATAGATCTAAACATTCTTAA


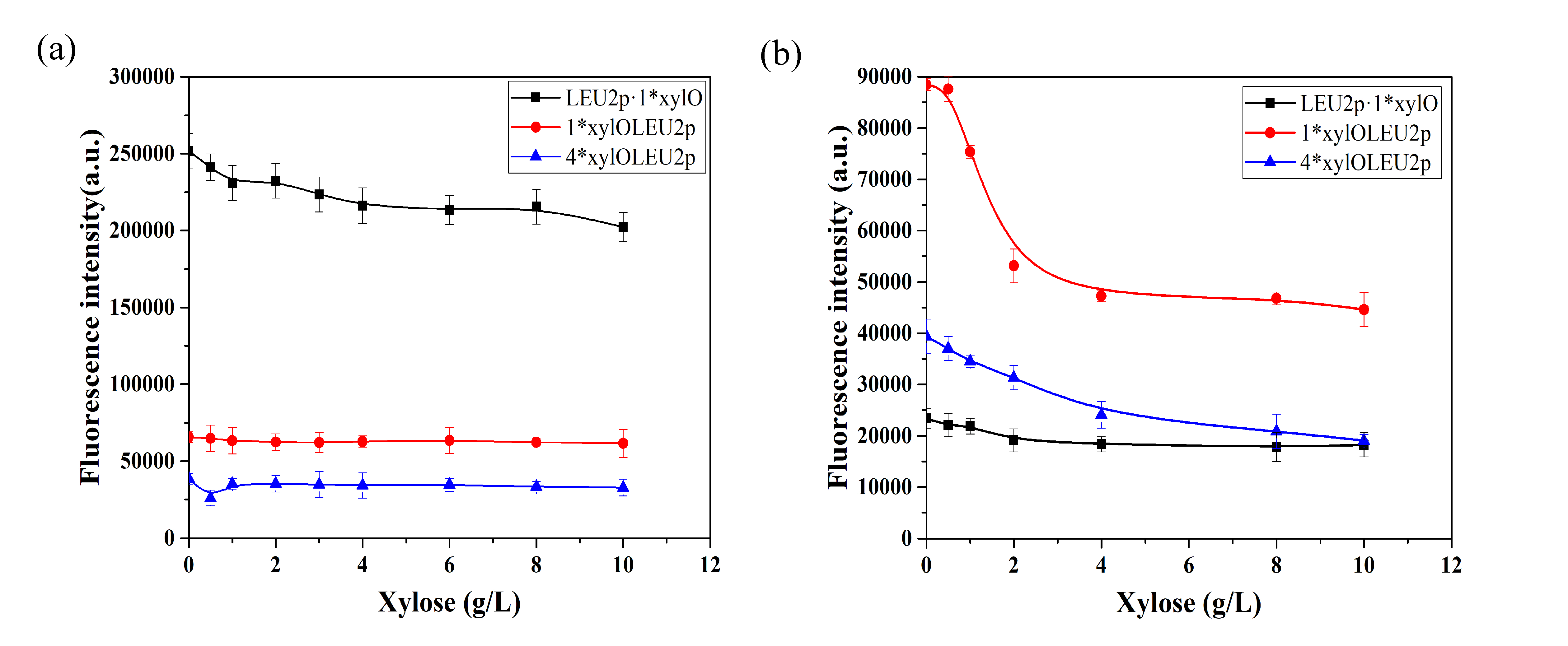


**Figure S1**: Characterization of xylose repression sensor in a minimal medium containing 2% glucose as the carbon source. (a) Dose-response curves of the strain containing “*LEU2p·1*xylO*”, “*1*xylOLEU2p*” and “*4*xylOLEU2p*” with XylR-Med2 in the presence of xylose. (b) Dose-response curves of the strain containing “*LEU2p·1*xylO*”, “*1*xylOLEU2p*” and “*4*xylOLEU2p*” with Med2-XylR in the presence of xylose.

References:

1. Chen Y, Daviet L, Schalk M, Siewers V, Nielsen J: **Establishing a platform cell factory through engineering of yeast acetyl-CoA metabolism.** *Metab Eng* 2013, **15:**48-54.
